# [2-(1-{2-[Aza­nid­yl(ethyl­sulfan­yl)methyl­idene-κ*N*]hydrazin-1-yl­idene-κ*N* ^1^}eth­yl)phenolato-κ*O*](dimethyl sulfoxide-*κO*)dioxidomolybdenum(VI)

**DOI:** 10.1107/S1600536812026566

**Published:** 2012-06-20

**Authors:** Reza Takjoo, Seik Weng Ng, Edward R. T. Tiekink

**Affiliations:** a Department of Chemistry, School of Sciences, Ferdowsi University of Mashhad, 91775-1436 Mashhad, Iran; b Department of Chemistry, University of Malaya, 50603 Kuala Lumpur, Malaysia; c Chemistry Department and Faculty of Science, King Abdulaziz University, PO Box 80203 Jeddah, Saudi Arabia

## Abstract

The Mo^VI^ atom in the title complex, [Mo(C_11_H_13_N_3_OS)O_2_(C_2_H_6_OS)], is *N*,*N*′,*O*-coordinated by the dianionic tridentate ligand, two mutually *cis* oxide O atoms and a dimethyl sulfoxide O atom, defining a distorted octa­hedral N_2_O_4_ donor set. The most prominent feature of the crystal packing is the formation of inversion dimers *via* pairs of N—H⋯O hydrogen bonds and eight-membered {⋯HNMoO}_2_ loops. The Schiff base ligand is disordered over two orientations of equal occupancy.

## Related literature

For the coordination chemistry and medicinal applications of thio­semicarbazone derivatives, see: Ahmadi *et al.* (2012 ▶); Dilworth & Hueting (2012 ▶). For related structures, see: Ceylan *et al.* (2009 ▶); Takjoo *et al.* (2012 ▶).
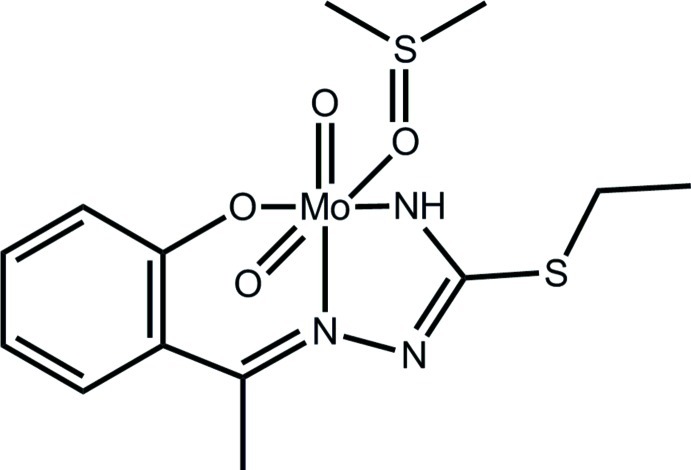


## Experimental

### 

#### Crystal data

[Mo(C_11_H_13_N_3_OS)O_2_(C_2_H_6_OS)]
*M* *_r_* = 441.37
Triclinic, 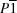

*a* = 8.0411 (3) Å
*b* = 9.7243 (3) Å
*c* = 12.0849 (4) Å
α = 73.387 (3)°
β = 84.465 (3)°
γ = 86.077 (3)°
*V* = 900.47 (5) Å^3^
*Z* = 2
Mo *K*α radiation
μ = 0.98 mm^−1^
*T* = 100 K
0.35 × 0.15 × 0.05 mm

#### Data collection

Agilent SuperNova Dual diffractometer with an Atlas detector
Absorption correction: multi-scan (*CrysAlis PRO*; Agilent, 2012 ▶) *T*_min_ = 0.691, *T*_max_ = 1.000
13626 measured reflections
4149 independent reflections
3811 reflections with *I* > 2σ(*I*)
*R*_int_ = 0.037

#### Refinement

*R*[*F* ^2^ > 2σ(*F* ^2^)] = 0.030
*wR*(*F* ^2^) = 0.072
*S* = 1.03
4149 reflections
234 parameters
100 restraints
H-atom parameters constrained
Δρ_max_ = 0.73 e Å^−3^
Δρ_min_ = −0.87 e Å^−3^

### 

Data collection: *CrysAlis PRO* (Agilent, 2012 ▶); cell refinement: *CrysAlis PRO*; data reduction: *CrysAlis PRO*; program(s) used to solve structure: *SHELXS97* (Sheldrick, 2008 ▶); program(s) used to refine structure: *SHELXL97* (Sheldrick, 2008 ▶); molecular graphics: *ORTEP-3* (Farrugia, 1997 ▶) and *DIAMOND* (Brandenburg, 2006 ▶); software used to prepare material for publication: *publCIF* (Westrip, 2010 ▶).

## Supplementary Material

Crystal structure: contains datablock(s) global, I. DOI: 10.1107/S1600536812026566/hb6848sup1.cif

Structure factors: contains datablock(s) I. DOI: 10.1107/S1600536812026566/hb6848Isup2.hkl

Additional supplementary materials:  crystallographic information; 3D view; checkCIF report

## Figures and Tables

**Table 1:** Selected bond lengths (Å)

Mo—O1	2.011 (7)
Mo—O2	2.2747 (16)
Mo—O3	1.7133 (16)
Mo—O4	1.7021 (18)
Mo—N1	2.193 (6)
Mo—N3	1.933 (15)

**Table 2:** Hydrogen-bond geometry (Å, °)

*D*—H⋯*A*	*D*—H	H⋯*A*	*D*⋯*A*	*D*—H⋯*A*
N3—H3*n*⋯O3^i^	0.88	2.23	3.090 (15)	166
N3′—H3*n*′⋯O3^i^	0.88	1.94	2.816 (16)	171

Symmetry code: (i) 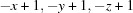.

**Fig. 1.**
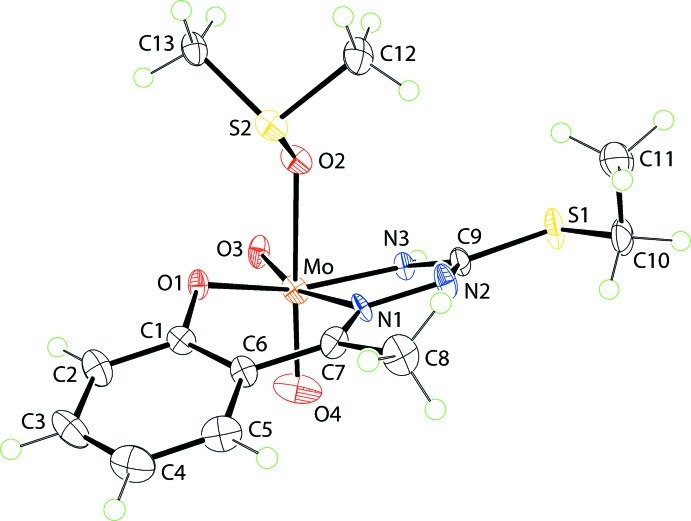
The molecular structure of (I) showing the atom-labelling scheme and displacement ellipsoids at the 50% probability level.

**Fig. 2.**
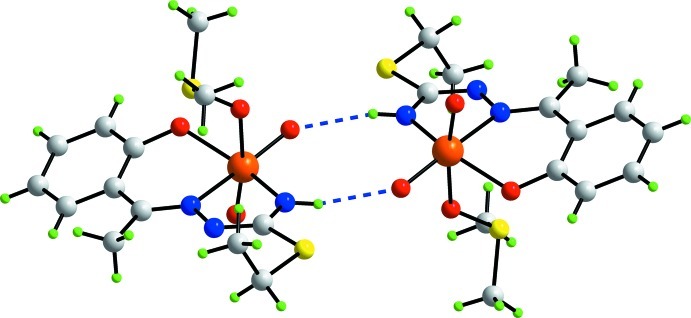
A view of the centrosymmetric aggregate in (I) mediated by N—H···O hydrogen bonds, shown as blue dashed lines. A similar arragement is found for the second conformation of the Schiff base ligand.

## supplementary crystallographic information

### Comment

Schiff bases derived from *S*-alkyl esters of thiosemicarbazone are capable of complexing both transition and main group metals (Ahmadi *et al.*, 2012) and these may be used as therapeutic and imaging agents (Dilworth & Hueting, 2012). Herein, the crystal and molecular structure of the title complex, (I), is described, which was determined as a part of on-going studies (Takjoo *et al.*, 2012).

The Mo^VI^ atom in (I), Fig. 1, exists within a distorted octahedral N_2_O_4_ donor set defined by the *N*,*N*,*O* atoms of the dinegative tridentate ligand, two oxo-O atoms and a DMSO-O atom, Table 1; the oxo-O atoms are *cis*. The dihedral angle between the five- and six-membered chelate rings is 11.34 (19) Å indicating some bending in the Schiff base ligand (the comparable angle in the second conformation of the disordered ligand is 7.81 (17)°). The molecular structure resembles that of the complex where the Mo atom is coordinated by methanol rather than DMSO (Ceylan *et al.*, 2009).

While whole molecule disorder of the Schiff base ligands precludes a detailed analysis of the crystal packing, a common feature of both orientations is the formation of N—H···O hydrogen bonds between centrosymmetrically related molecules to form a dimeric aggregate *via* an eight-membered {···HNMoO}_2_ synthon, Fig. 2 and Table 2.

### Experimental

An ethanolic solution (3 ml) of molybdenyl acetylacetonate (0.33 g, 1 mmol) was added drop-wise to an ethanolic solution (3 ml) of 1-(2-hydroxyphenyl)ethanone *S*-ethylisothiosemicarbazone hydrobromide (0.32 g, 1 mmol) under stirring. The clear solution was stirred for 1 h and yellow precipitate was appeared. The product was then filtered, washed with cold ethanol and dried in air. The resulting compound was dissolved in DMSO (2 ml) and by slow evaporation of the solvent orange prisms appeared after one week. *M*.pt. 427 K. Yield: 33%.

### Refinement

Nitrogen- and carbon-bound H-atoms were placed in calculated positions [N—H = 0.88 Å and C—H = 0.95–0.99 Å, *U*_iso_(H) = 1.2–1.5*U*_eq_(N,*C*)] and were included in the refinement in the riding model approximation.

The dianion is disordered over two positions in a 1:1 ratio. The benzene rings were refined as rigid hexagons of 1.39 Å sides and other pairs of bond distances were restrained to within 0.01 Å of each other. The anisotropic displacement parameters (restrained to be nearly isotropic) of the primed atoms were set to those of the unprimed ones.

### Crystal data

**Table:**

[Mo(C_11_H_13_N_3_OS)O_2_(C_2_H_6_OS)]	*Z* = 2
*M**_r_* = 441.37	*F*(000) = 448
Triclinic, *P*1	*D*_x_ = 1.628 Mg m^−^^3^
Hall symbol: -P 1	Mo *K*α radiation, λ = 0.71073 Å
*a* = 8.0411 (3) Å	Cell parameters from 8498 reflections
*b* = 9.7243 (3) Å	θ = 2.4–27.5°
*c* = 12.0849 (4) Å	µ = 0.98 mm^−^^1^
α = 73.387 (3)°	*T* = 100 K
β = 84.465 (3)°	Prism, orange
γ = 86.077 (3)°	0.35 × 0.15 × 0.05 mm
*V* = 900.47 (5) Å^3^	

### Data collection

**Table:**

Agilent SuperNova Dual diffractometer with an Atlas detector	4149 independent reflections
Radiation source: SuperNova (Mo) X-ray Source	3811 reflections with *I* > 2σ(*I*)
Mirror monochromator	*R*_int_ = 0.037
Detector resolution: 10.4041 pixels mm^-1^	θ_max_ = 27.6°, θ_min_ = 2.4°
ω scan	*h* = −10→10
Absorption correction: multi-scan (*CrysAlis PRO*; Agilent, 2012)	*k* = −12→12
*T*_min_ = 0.691, *T*_max_ = 1.000	*l* = −15→15
13626 measured reflections	

### Refinement

**Table:**

Refinement on *F*^2^	Primary atom site location: structure-invariant direct methods
Least-squares matrix: full	Secondary atom site location: difference Fourier map
*R*[*F*^2^ > 2σ(*F*^2^)] = 0.030	Hydrogen site location: inferred from neighbouring sites
*wR*(*F*^2^) = 0.072	H-atom parameters constrained
*S* = 1.03	*w* = 1/[σ^2^(*F*_o_^2^) + (0.0278*P*)^2^ + 1.0887*P*] where *P* = (*F*_o_^2^ + 2*F*_c_^2^)/3
4149 reflections	(Δ/σ)_max_ = 0.001
234 parameters	Δρ_max_ = 0.73 e Å^−^^3^
100 restraints	Δρ_min_ = −0.87 e Å^−^^3^

### Special details

**Table:**

Geometry. All e.s.d.'s (except the e.s.d. in the dihedral angle between two l.s. planes) are estimated using the full covariance matrix. The cell e.s.d.'s are taken into account individually in the estimation of e.s.d.'s in distances, angles and torsion angles; correlations between e.s.d.'s in cell parameters are only used when they are defined by crystal symmetry. An approximate (isotropic) treatment of cell e.s.d.'s is used for estimating e.s.d.'s involving l.s. planes.
Refinement. Refinement of *F*^2^ against ALL reflections. The weighted *R*-factor *wR* and goodness of fit *S* are based on *F*^2^, conventional *R*-factors *R* are based on *F*, with *F* set to zero for negative *F*^2^. The threshold expression of *F*^2^ > σ(*F*^2^) is used only for calculating *R*-factors(gt) *etc*. and is not relevant to the choice of reflections for refinement. *R*-factors based on *F*^2^ are statistically about twice as large as those based on *F*, and *R*- factors based on ALL data will be even larger.

### Fractional atomic coordinates and isotropic or equivalent isotropic displacement parameters (Å^2^)

**Table:**

	*x*	*y*	*z*	*U*_iso_*/*U*_eq_	Occ. (<1)
Mo	0.39828 (2)	0.62362 (2)	0.284368 (16)	0.01779 (7)	
S2	−0.02453 (7)	0.59823 (6)	0.30257 (5)	0.02079 (13)	
O2	0.13555 (19)	0.56941 (17)	0.36645 (13)	0.0208 (3)	
O3	0.4236 (2)	0.67549 (16)	0.40557 (15)	0.0217 (3)	
O4	0.5828 (2)	0.6597 (2)	0.20227 (15)	0.0370 (5)	
C12	−0.1234 (3)	0.4305 (3)	0.3508 (2)	0.0268 (5)	
H12A	−0.0630	0.3620	0.3141	0.040*	
H12B	−0.2393	0.4441	0.3296	0.040*	
H12C	−0.1223	0.3934	0.4351	0.040*	
C13	−0.1570 (3)	0.6982 (2)	0.3810 (2)	0.0221 (5)	
H13A	−0.1176	0.7959	0.3631	0.033*	
H13B	−0.1547	0.6515	0.4643	0.033*	
H13C	−0.2717	0.7022	0.3588	0.033*	
S1	0.4023 (2)	0.14457 (15)	0.38075 (13)	0.0307 (3)	0.50
O1	0.2528 (12)	0.7850 (6)	0.1938 (4)	0.0162 (9)	0.50
N1	0.3150 (11)	0.5172 (5)	0.1633 (5)	0.0149 (10)	0.50
N2	0.3133 (9)	0.3667 (6)	0.2053 (4)	0.0203 (9)	0.50
N3	0.439 (3)	0.4201 (15)	0.3527 (10)	0.0137 (16)	0.50
H3n	0.4952	0.3893	0.4149	0.016*	0.50
C1	0.2566 (5)	0.8284 (3)	0.07631 (19)	0.0176 (9)	0.50
C2	0.2480 (5)	0.9757 (3)	0.0241 (3)	0.0275 (8)	0.50
H2	0.2412	1.0405	0.0703	0.033*	0.50
C3	0.2494 (5)	1.0281 (3)	−0.0957 (3)	0.0329 (9)	0.50
H3	0.2435	1.1287	−0.1314	0.040*	0.50
C4	0.2593 (5)	0.9332 (4)	−0.16331 (19)	0.0335 (9)	0.50
H4	0.2602	0.9691	−0.2452	0.040*	0.50
C5	0.2679 (5)	0.7860 (3)	−0.1111 (3)	0.0278 (8)	0.50
H5	0.2747	0.7211	−0.1573	0.033*	0.50
C6	0.2666 (4)	0.7335 (2)	0.0087 (3)	0.0183 (8)	0.50
C7	0.2706 (7)	0.5771 (6)	0.0576 (4)	0.0188 (9)	0.50
C8	0.2263 (7)	0.4788 (6)	−0.0121 (4)	0.0277 (8)	0.50
H8A	0.1657	0.3976	0.0391	0.042*	0.50
H8B	0.3291	0.4431	−0.0472	0.042*	0.50
H8C	0.1557	0.5326	−0.0732	0.042*	0.50
C9	0.3817 (12)	0.3257 (6)	0.3040 (5)	0.0185 (11)	0.50
C10	0.3280 (7)	0.0531 (6)	0.2844 (5)	0.0306 (9)	0.50
H10A	0.3694	0.1019	0.2038	0.037*	0.50
H10B	0.3759	−0.0467	0.3039	0.037*	0.50
C11	0.1370 (8)	0.0490 (7)	0.2911 (6)	0.0422 (12)	0.50
H11A	0.1011	0.0621	0.2134	0.063*	0.50
H11B	0.0866	0.1263	0.3222	0.063*	0.50
H11C	0.1013	−0.0439	0.3419	0.063*	0.50
S1'	0.3906 (2)	0.12666 (15)	0.43708 (13)	0.0307 (3)	0.50
O1'	0.2699 (12)	0.7538 (6)	0.1728 (4)	0.0162 (9)	0.50
N1'	0.3129 (11)	0.4692 (6)	0.1856 (5)	0.0149 (10)	0.50
N2'	0.3038 (9)	0.3236 (6)	0.2459 (4)	0.0203 (9)	0.50
N3'	0.434 (3)	0.4047 (15)	0.3800 (10)	0.0137 (16)	0.50
H3n'	0.4895	0.3818	0.4426	0.016*	0.50
C1'	0.2776 (5)	0.7739 (3)	0.05697 (19)	0.0176 (9)	0.50
C2'	0.2822 (5)	0.9143 (3)	−0.0140 (3)	0.0275 (8)	0.50
H2'	0.2816	0.9919	0.0193	0.033*	0.50
C3'	0.2878 (5)	0.9413 (3)	−0.1336 (3)	0.0329 (9)	0.50
H3'	0.2910	1.0372	−0.1821	0.040*	0.50
C4'	0.2888 (5)	0.8278 (4)	−0.18223 (19)	0.0335 (9)	0.50
H4'	0.2926	0.8462	−0.2640	0.040*	0.50
C5'	0.2841 (5)	0.6874 (3)	−0.1113 (3)	0.0278 (8)	0.50
H5'	0.2847	0.6099	−0.1445	0.033*	0.50
C6'	0.2785 (5)	0.6605 (3)	0.0083 (2)	0.0183 (8)	0.50
C7'	0.2681 (7)	0.5090 (6)	0.0789 (5)	0.0188 (9)	0.50
C8'	0.2100 (7)	0.3937 (6)	0.0307 (4)	0.0277 (8)	0.50
H8'A	0.1395	0.3280	0.0906	0.042*	0.50
H8'B	0.3076	0.3399	0.0065	0.042*	0.50
H8'C	0.1456	0.4388	−0.0362	0.042*	0.50
C9'	0.3720 (12)	0.3009 (6)	0.3435 (5)	0.0185 (11)	0.50
C10'	0.3169 (7)	0.0189 (5)	0.3535 (5)	0.0306 (9)	0.50
H10C	0.3611	0.0564	0.2717	0.037*	0.50
H10D	0.3621	−0.0809	0.3826	0.037*	0.50
C11'	0.1275 (8)	0.0176 (7)	0.3585 (6)	0.0422 (12)	0.50
H11D	0.0958	−0.0464	0.3151	0.063*	0.50
H11E	0.0821	0.1151	0.3241	0.063*	0.50
H11F	0.0823	−0.0167	0.4394	0.063*	0.50

### Atomic displacement parameters (Å^2^)

**Table:**

	*U*^11^	*U*^22^	*U*^33^	*U*^12^	*U*^13^	*U*^23^
Mo	0.01666 (11)	0.01953 (11)	0.01794 (11)	−0.00370 (7)	−0.00961 (7)	−0.00315 (8)
S2	0.0173 (3)	0.0254 (3)	0.0192 (3)	−0.0038 (2)	−0.0098 (2)	−0.0022 (2)
O2	0.0160 (8)	0.0250 (8)	0.0199 (8)	−0.0045 (6)	−0.0099 (6)	−0.0005 (7)
O3	0.0200 (8)	0.0159 (8)	0.0345 (9)	0.0010 (6)	−0.0119 (7)	−0.0130 (7)
O4	0.0225 (9)	0.0648 (14)	0.0226 (9)	−0.0156 (9)	−0.0076 (7)	−0.0053 (9)
C12	0.0229 (12)	0.0233 (12)	0.0397 (14)	−0.0036 (10)	−0.0110 (10)	−0.0141 (11)
C13	0.0203 (11)	0.0164 (11)	0.0312 (12)	−0.0030 (9)	−0.0110 (9)	−0.0056 (9)
S1	0.0580 (6)	0.0097 (4)	0.0242 (6)	0.0007 (4)	−0.0099 (8)	−0.0029 (6)
O1	0.0220 (19)	0.012 (2)	0.0160 (17)	0.0021 (19)	−0.0077 (17)	−0.0041 (12)
N1	0.0204 (11)	0.008 (3)	0.015 (2)	−0.006 (3)	−0.0055 (18)	0.000 (2)
N2	0.0332 (15)	0.012 (3)	0.017 (3)	−0.005 (2)	−0.010 (2)	−0.0030 (19)
N3	0.0215 (15)	0.012 (2)	0.008 (5)	0.000 (2)	−0.009 (5)	−0.002 (3)
C1	0.0167 (15)	0.021 (2)	0.0157 (15)	−0.0021 (17)	−0.0070 (12)	−0.0037 (15)
C2	0.031 (2)	0.021 (2)	0.028 (2)	−0.0037 (16)	−0.0094 (16)	−0.0009 (13)
C3	0.036 (2)	0.028 (2)	0.028 (2)	−0.0096 (17)	−0.0079 (16)	0.0077 (15)
C4	0.029 (2)	0.045 (2)	0.0224 (18)	−0.0022 (18)	−0.0092 (15)	−0.0005 (18)
C5	0.0245 (17)	0.039 (2)	0.0215 (15)	−0.0002 (18)	−0.0089 (13)	−0.0087 (18)
C6	0.0172 (14)	0.017 (2)	0.0203 (14)	−0.0016 (19)	−0.0079 (11)	−0.003 (2)
C7	0.0164 (13)	0.021 (3)	0.025 (2)	−0.003 (2)	−0.0070 (14)	−0.014 (2)
C8	0.037 (2)	0.031 (2)	0.0202 (19)	−0.0053 (19)	−0.0133 (16)	−0.0112 (15)
C9	0.0272 (17)	0.011 (2)	0.018 (4)	−0.0034 (18)	−0.002 (3)	−0.004 (2)
C10	0.046 (2)	0.0160 (18)	0.034 (2)	−0.0063 (15)	0.000 (2)	−0.0140 (19)
C11	0.048 (2)	0.034 (3)	0.052 (3)	−0.0188 (19)	0.012 (3)	−0.026 (3)
S1'	0.0580 (6)	0.0097 (4)	0.0242 (6)	0.0007 (4)	−0.0099 (8)	−0.0029 (6)
O1'	0.0220 (19)	0.012 (2)	0.0160 (17)	0.0021 (19)	−0.0077 (17)	−0.0041 (12)
N1'	0.0204 (11)	0.008 (3)	0.015 (2)	−0.006 (3)	−0.0055 (18)	0.000 (2)
N2'	0.0332 (15)	0.012 (3)	0.017 (3)	−0.005 (2)	−0.010 (2)	−0.0030 (19)
N3'	0.0215 (15)	0.012 (2)	0.008 (5)	0.000 (2)	−0.009 (5)	−0.002 (3)
C1'	0.0167 (15)	0.021 (2)	0.0157 (15)	−0.0021 (17)	−0.0070 (12)	−0.0037 (15)
C2'	0.031 (2)	0.021 (2)	0.028 (2)	−0.0037 (16)	−0.0094 (16)	−0.0009 (13)
C3'	0.036 (2)	0.028 (2)	0.028 (2)	−0.0096 (17)	−0.0079 (16)	0.0077 (15)
C4'	0.029 (2)	0.045 (2)	0.0224 (18)	−0.0022 (18)	−0.0092 (15)	−0.0005 (18)
C5'	0.0245 (17)	0.039 (2)	0.0215 (15)	−0.0002 (18)	−0.0089 (13)	−0.0087 (18)
C6'	0.0172 (14)	0.017 (2)	0.0203 (14)	−0.0016 (19)	−0.0079 (11)	−0.003 (2)
C7'	0.0164 (13)	0.021 (3)	0.025 (2)	−0.003 (2)	−0.0070 (14)	−0.014 (2)
C8'	0.037 (2)	0.031 (2)	0.0202 (19)	−0.0053 (19)	−0.0133 (16)	−0.0112 (15)
C9'	0.0272 (17)	0.011 (2)	0.018 (4)	−0.0034 (18)	−0.002 (3)	−0.004 (2)
C10'	0.046 (2)	0.0160 (18)	0.034 (2)	−0.0063 (15)	0.000 (2)	−0.0140 (19)
C11'	0.048 (2)	0.034 (3)	0.052 (3)	−0.0188 (19)	0.012 (3)	−0.026 (3)

### Geometric parameters (Å, º)

**Table:**

Mo—O1	2.011 (7)	C8—H8A	0.9800
Mo—O2	2.2747 (16)	C8—H8B	0.9800
Mo—O3	1.7133 (16)	C8—H8C	0.9800
Mo—O4	1.7021 (18)	C10—C11	1.533 (7)
Mo—O1'	1.897 (7)	C10—H10A	0.9900
Mo—N1	2.193 (6)	C10—H10B	0.9900
Mo—N3	1.933 (15)	C11—H11A	0.9800
Mo—N3'	2.127 (15)	C11—H11B	0.9800
Mo—N1'	2.340 (6)	C11—H11C	0.9800
S2—O2	1.5330 (15)	S1'—C9'	1.753 (5)
S2—C13	1.780 (2)	S1'—C10'	1.810 (5)
S2—C12	1.782 (2)	O1'—C1'	1.353 (5)
C12—H12A	0.9800	N1'—C7'	1.315 (6)
C12—H12B	0.9800	N1'—N2'	1.398 (5)
C12—H12C	0.9800	N2'—C9'	1.304 (6)
C13—H13A	0.9800	N3'—C9'	1.353 (6)
C13—H13B	0.9800	N3'—H3n'	0.8800
C13—H13C	0.9800	C1'—C2'	1.3900
S1—C9	1.746 (6)	C1'—C6'	1.3900
S1—C10	1.818 (5)	C2'—C3'	1.3900
O1—C1	1.359 (5)	C2'—H2'	0.9500
N1—C7	1.316 (6)	C3'—C4'	1.3900
N1—N2	1.406 (5)	C3'—H3'	0.9500
N2—C9	1.307 (6)	C4'—C5'	1.3900
N3—C9	1.351 (6)	C4'—H4'	0.9500
N3—H3n	0.8800	C5'—C6'	1.3900
C1—C2	1.3900	C5'—H5'	0.9500
C1—C6	1.3900	C6'—C7'	1.479 (5)
C2—C3	1.3900	C7'—C8'	1.521 (6)
C2—H2	0.9500	C8'—H8'A	0.9800
C3—C4	1.3900	C8'—H8'B	0.9800
C3—H3	0.9500	C8'—H8'C	0.9800
C4—C5	1.3900	C10'—C11'	1.519 (7)
C4—H4	0.9500	C10'—H10C	0.9900
C5—C6	1.3900	C10'—H10D	0.9900
C5—H5	0.9500	C11'—H11D	0.9800
C6—C7	1.465 (6)	C11'—H11E	0.9800
C7—C8	1.522 (6)	C11'—H11F	0.9800
			
O4—Mo—O3	104.39 (8)	C6—C5—H5	120.0
O4—Mo—O1'	94.2 (3)	C4—C5—H5	120.0
O3—Mo—O1'	115.50 (14)	C5—C6—C1	120.0
O4—Mo—N3	98.5 (7)	C5—C6—C7	116.8 (3)
O3—Mo—N3	96.7 (3)	C1—C6—C7	123.2 (3)
O1'—Mo—N3	141.2 (5)	N1—C7—C6	120.7 (4)
O4—Mo—O1	99.5 (3)	N1—C7—C8	117.9 (5)
O3—Mo—O1	102.65 (14)	C6—C7—C8	121.4 (4)
O1'—Mo—O1	12.9 (2)	N2—C9—N3	122.3 (8)
N3—Mo—O1	149.3 (6)	N2—C9—S1	121.5 (5)
O4—Mo—N3'	103.0 (6)	N3—C9—S1	116.2 (8)
O3—Mo—N3'	90.1 (3)	C11—C10—S1	113.6 (4)
O1'—Mo—N3'	144.7 (5)	C11—C10—H10A	108.9
N3—Mo—N3'	7.2 (8)	S1—C10—H10A	108.9
O1—Mo—N3'	150.4 (6)	C11—C10—H10B	108.9
O4—Mo—N1	90.4 (2)	S1—C10—H10B	108.9
O3—Mo—N1	163.71 (19)	H10A—C10—H10B	107.7
O1'—Mo—N1	69.3 (2)	C10—C11—H11A	109.5
N3—Mo—N1	74.0 (3)	C10—C11—H11B	109.5
O1—Mo—N1	81.2 (2)	H11A—C11—H11B	109.5
N3'—Mo—N1	79.8 (3)	C10—C11—H11C	109.5
O4—Mo—O2	170.16 (7)	H11A—C11—H11C	109.5
O3—Mo—O2	85.28 (7)	H11B—C11—H11C	109.5
O1'—Mo—O2	79.7 (3)	C9'—S1'—C10'	102.2 (3)
N3—Mo—O2	82.0 (7)	C1'—O1'—Mo	128.9 (4)
O1—Mo—O2	76.2 (3)	C7'—N1'—N2'	117.3 (5)
N3'—Mo—O2	78.5 (6)	C7'—N1'—Mo	125.2 (4)
N1—Mo—O2	80.3 (2)	N2'—N1'—Mo	117.5 (4)
O4—Mo—N1'	94.7 (2)	C9'—N2'—N1'	108.8 (5)
O3—Mo—N1'	154.27 (16)	C9'—N3'—Mo	119.4 (9)
O1'—Mo—N1'	79.4 (2)	C9'—N3'—H3n'	120.3
N3—Mo—N1'	63.1 (3)	Mo—N3'—H3n'	120.3
O1—Mo—N1'	90.7 (2)	O1'—C1'—C2'	117.6 (3)
N3'—Mo—N1'	68.7 (3)	O1'—C1'—C6'	122.4 (3)
N1—Mo—N1'	11.27 (17)	C2'—C1'—C6'	120.0
O2—Mo—N1'	76.6 (2)	C1'—C2'—C3'	120.0
O2—S2—C13	103.33 (10)	C1'—C2'—H2'	120.0
O2—S2—C12	103.52 (10)	C3'—C2'—H2'	120.0
C13—S2—C12	99.67 (12)	C4'—C3'—C2'	120.0
S2—O2—Mo	125.84 (9)	C4'—C3'—H3'	120.0
S2—C12—H12A	109.5	C2'—C3'—H3'	120.0
S2—C12—H12B	109.5	C3'—C4'—C5'	120.0
H12A—C12—H12B	109.5	C3'—C4'—H4'	120.0
S2—C12—H12C	109.5	C5'—C4'—H4'	120.0
H12A—C12—H12C	109.5	C6'—C5'—C4'	120.0
H12B—C12—H12C	109.5	C6'—C5'—H5'	120.0
S2—C13—H13A	109.5	C4'—C5'—H5'	120.0
S2—C13—H13B	109.5	C5'—C6'—C1'	120.0
H13A—C13—H13B	109.5	C5'—C6'—C7'	117.2 (3)
S2—C13—H13C	109.5	C1'—C6'—C7'	122.7 (3)
H13A—C13—H13C	109.5	N1'—C7'—C6'	120.5 (4)
H13B—C13—H13C	109.5	N1'—C7'—C8'	117.6 (5)
C9—S1—C10	103.3 (3)	C6'—C7'—C8'	121.8 (4)
C1—O1—Mo	124.2 (4)	C7'—C8'—H8'A	109.5
C7—N1—N2	117.6 (5)	C7'—C8'—H8'B	109.5
C7—N1—Mo	127.8 (4)	H8'A—C8'—H8'B	109.5
N2—N1—Mo	114.5 (4)	C7'—C8'—H8'C	109.5
C9—N2—N1	108.9 (5)	H8'A—C8'—H8'C	109.5
C9—N3—Mo	119.3 (10)	H8'B—C8'—H8'C	109.5
C9—N3—H3n	120.3	N2'—C9'—N3'	124.3 (8)
Mo—N3—H3n	120.3	N2'—C9'—S1'	120.4 (5)
O1—C1—C2	116.7 (3)	N3'—C9'—S1'	115.2 (7)
O1—C1—C6	123.3 (3)	C11'—C10'—S1'	113.5 (4)
C2—C1—C6	120.0	C11'—C10'—H10C	108.9
C3—C2—C1	120.0	S1'—C10'—H10C	108.9
C3—C2—H2	120.0	C11'—C10'—H10D	108.9
C1—C2—H2	120.0	S1'—C10'—H10D	108.9
C4—C3—C2	120.0	H10C—C10'—H10D	107.7
C4—C3—H3	120.0	C10'—C11'—H11D	109.5
C2—C3—H3	120.0	C10'—C11'—H11E	109.5
C3—C4—C5	120.0	H11D—C11'—H11E	109.5
C3—C4—H4	120.0	C10'—C11'—H11F	109.5
C5—C4—H4	120.0	H11D—C11'—H11F	109.5
C6—C5—C4	120.0	H11E—C11'—H11F	109.5
			
C13—S2—O2—Mo	−125.78 (12)	Mo—N3—C9—S1	−172.6 (9)
C12—S2—O2—Mo	130.66 (13)	C10—S1—C9—N2	4.7 (8)
O3—Mo—O2—S2	139.32 (13)	C10—S1—C9—N3	−175.7 (12)
O1'—Mo—O2—S2	22.32 (18)	C9—S1—C10—C11	−81.4 (5)
N3—Mo—O2—S2	−123.3 (3)	O4—Mo—O1'—C1'	44.6 (7)
O1—Mo—O2—S2	35.04 (18)	O3—Mo—O1'—C1'	152.7 (6)
N3'—Mo—O2—S2	−129.7 (3)	N3—Mo—O1'—C1'	−64.4 (14)
N1—Mo—O2—S2	−48.22 (18)	O1—Mo—O1'—C1'	160 (3)
N1'—Mo—O2—S2	−59.11 (17)	N3'—Mo—O1'—C1'	−74.9 (13)
O4—Mo—O1—C1	42.4 (7)	N1—Mo—O1'—C1'	−44.2 (7)
O3—Mo—O1—C1	149.6 (6)	O2—Mo—O1'—C1'	−127.6 (8)
O1'—Mo—O1—C1	−23.9 (18)	N1'—Mo—O1'—C1'	−49.4 (7)
N3—Mo—O1—C1	−82.8 (13)	O4—Mo—N1'—C7'	−69.8 (7)
N3'—Mo—O1—C1	−97.0 (11)	O3—Mo—N1'—C7'	151.9 (4)
N1—Mo—O1—C1	−46.5 (7)	O1'—Mo—N1'—C7'	23.5 (7)
O2—Mo—O1—C1	−128.6 (7)	N3—Mo—N1'—C7'	−166.9 (11)
N1'—Mo—O1—C1	−52.6 (7)	O1—Mo—N1'—C7'	29.8 (8)
O4—Mo—N1—C7	−71.6 (8)	N3'—Mo—N1'—C7'	−171.9 (11)
O3—Mo—N1—C7	133.1 (5)	N1—Mo—N1'—C7'	−2.3 (17)
O1'—Mo—N1—C7	22.7 (7)	O2—Mo—N1'—C7'	105.4 (7)
N3—Mo—N1—C7	−170.3 (11)	O4—Mo—N1'—N2'	112.3 (6)
O1—Mo—N1—C7	28.0 (8)	O3—Mo—N1'—N2'	−26.0 (10)
N3'—Mo—N1—C7	−174.7 (10)	O1'—Mo—N1'—N2'	−154.4 (7)
O2—Mo—N1—C7	105.3 (8)	N3—Mo—N1'—N2'	15.2 (9)
N1'—Mo—N1—C7	175 (3)	O1—Mo—N1'—N2'	−148.1 (7)
O4—Mo—N1—N2	107.1 (6)	N3'—Mo—N1'—N2'	10.2 (9)
O3—Mo—N1—N2	−48.2 (12)	N1—Mo—N1'—N2'	180 (3)
O1'—Mo—N1—N2	−158.6 (7)	O2—Mo—N1'—N2'	−72.5 (6)
N3—Mo—N1—N2	8.4 (9)	C7'—N1'—N2'—C9'	172.1 (8)
O1—Mo—N1—N2	−153.3 (7)	Mo—N1'—N2'—C9'	−9.8 (9)
N3'—Mo—N1—N2	4.0 (9)	O4—Mo—N3'—C9'	−98.7 (15)
O2—Mo—N1—N2	−76.0 (6)	O3—Mo—N3'—C9'	156.5 (15)
N1'—Mo—N1—N2	−5.9 (18)	O1'—Mo—N3'—C9'	18 (2)
C7—N1—N2—C9	171.6 (8)	N3—Mo—N3'—C9'	−47 (8)
Mo—N1—N2—C9	−7.2 (9)	O1—Mo—N3'—C9'	40 (2)
O4—Mo—N3—C9	−95.9 (15)	N1—Mo—N3'—C9'	−10.7 (14)
O3—Mo—N3—C9	158.4 (14)	O2—Mo—N3'—C9'	71.4 (15)
O1'—Mo—N3—C9	12 (2)	N1'—Mo—N3'—C9'	−8.6 (13)
O1—Mo—N3—C9	30 (2)	Mo—O1'—C1'—C2'	−132.5 (5)
N3'—Mo—N3—C9	135 (10)	Mo—O1'—C1'—C6'	48.3 (9)
N1—Mo—N3—C9	−7.9 (13)	O1'—C1'—C2'—C3'	−179.1 (6)
O2—Mo—N3—C9	74.2 (15)	C6'—C1'—C2'—C3'	0.0
N1'—Mo—N3—C9	−4.8 (12)	C1'—C2'—C3'—C4'	0.0
Mo—O1—C1—C2	−138.2 (4)	C2'—C3'—C4'—C5'	0.0
Mo—O1—C1—C6	42.6 (9)	C3'—C4'—C5'—C6'	0.0
O1—C1—C2—C3	−179.3 (6)	C4'—C5'—C6'—C1'	0.0
C6—C1—C2—C3	0.0	C4'—C5'—C6'—C7'	178.0 (4)
C1—C2—C3—C4	0.0	O1'—C1'—C6'—C5'	179.1 (6)
C2—C3—C4—C5	0.0	C2'—C1'—C6'—C5'	0.0
C3—C4—C5—C6	0.0	O1'—C1'—C6'—C7'	1.2 (6)
C4—C5—C6—C1	0.0	C2'—C1'—C6'—C7'	−177.9 (4)
C4—C5—C6—C7	178.1 (4)	N2'—N1'—C7'—C6'	−178.4 (6)
O1—C1—C6—C5	179.2 (6)	Mo—N1'—C7'—C6'	3.7 (10)
C2—C1—C6—C5	0.0	N2'—N1'—C7'—C8'	0.1 (11)
O1—C1—C6—C7	1.3 (6)	Mo—N1'—C7'—C8'	−177.8 (5)
C2—C1—C6—C7	−178.0 (4)	C5'—C6'—C7'—N1'	159.1 (6)
N2—N1—C7—C6	179.0 (6)	C1'—C6'—C7'—N1'	−23.0 (8)
Mo—N1—C7—C6	−2.4 (11)	C5'—C6'—C7'—C8'	−19.4 (6)
N2—N1—C7—C8	−2.0 (11)	C1'—C6'—C7'—C8'	158.6 (4)
Mo—N1—C7—C8	176.7 (5)	N1'—N2'—C9'—N3'	2.3 (17)
C5—C6—C7—N1	161.3 (6)	N1'—N2'—C9'—S1'	−176.8 (6)
C1—C6—C7—N1	−20.6 (8)	Mo—N3'—C9'—N2'	7 (2)
C5—C6—C7—C8	−17.7 (6)	Mo—N3'—C9'—S1'	−173.7 (8)
C1—C6—C7—C8	160.3 (4)	C10'—S1'—C9'—N2'	5.2 (8)
N1—N2—C9—N3	0.9 (16)	C10'—S1'—C9'—N3'	−174.0 (12)
N1—N2—C9—S1	−179.5 (6)	C9'—S1'—C10'—C11'	−80.7 (5)
Mo—N3—C9—N2	7 (2)		

### Hydrogen-bond geometry (Å, º)

**Table:**

*D*—H···*A*	*D*—H	H···*A*	*D*···*A*	*D*—H···*A*
N3—H3*n*···O3^i^	0.88	2.23	3.090 (15)	166
N3′—H3*n*′···O3^i^	0.88	1.94	2.816 (16)	171

Symmetry code: (i) −*x*+1, −*y*+1, −*z*+1.
